# Stable coexistence of genetically divergent Atlantic cod ecotypes at multiple spatial scales

**DOI:** 10.1111/eva.12640

**Published:** 2018-05-17

**Authors:** Halvor Knutsen, Per Erik Jorde, Jeffrey A. Hutchings, Jakob Hemmer‐Hansen, Peter Grønkjær, Kris‐Emil Mose Jørgensen, Carl André, Marte Sodeland, Jon Albretsen, Esben M. Olsen

**Affiliations:** ^1^ Institute of Marine Research Flødevigen His Norway; ^2^ Department of Biosciences Centre for Ecological and Evolutionary Synthesis (CEES) University of Oslo Blindern Oslo Norway; ^3^ Centre for Coastal Research University of Agder Kristiansand Norway; ^4^ Department of Biology Dalhousie University Halifax NS Canada; ^5^ Section for Marine Living Resources National Institute of Aquatic Resources Technical University of Denmark Silkeborg Denmark; ^6^ Department for Bioscience Aarhus University Aquatic Biology Aarhus Denmark; ^7^ Department of Marine Sciences – Tjärnö University of Gothenburg Strömstad Sweden

**Keywords:** Atlantic cod, connectivity, dispersal, ecotypes, natural selection, sympatry, temporal genetic stability

## Abstract

Coexistence in the same habitat of closely related yet genetically different populations is a phenomenon that challenges our understanding of local population structure and adaptation. Identifying the underlying mechanisms for such coexistence can yield new insight into adaptive evolution, diversification and the potential for organisms to adapt and persist in response to a changing environment. Recent studies have documented cryptic, sympatric populations of Atlantic cod (*Gadus morhua*) in coastal areas. We analysed genetic origin of 6,483 individual cod sampled annually over 14 years from 125 locations along the Norwegian Skagerrak coast and document stable coexistence of two genetically divergent Atlantic cod ecotypes throughout the study area and study period. A “fjord” ecotype dominated in numbers deep inside fjords while a “North Sea” ecotype was the only type found in offshore North Sea. Both ecotypes coexisted in similar proportions throughout coastal habitats at all spatial scales. The size‐at‐age of the North Sea ecotype on average exceeded that of the fjord ecotype by 20% in length and 80% in weight across all habitats. Different growth and size among individuals of the two types might be one of several ecologically significant variables that allow for stable coexistence of closely related populations within the same habitat. Management plans, biodiversity initiatives and other mitigation strategies that do not account for the mixture of species ecotypes are unlikely to meet objectives related to the sustainability of fish and fisheries.

## 1 INTRODUCTION

Local populations are expected to evolve adaptations to their respective environmental conditions in the absence of other constraints (Ciannelli, Bailey, & Olsen, 2015), leading to resident genotypes having a higher relative fitness in their local habitat than genotypes originating from other habitats (Kawecki & Ebert, 2004). These fitness differences should favour local genotypes at the expense of other conspecifics and may result in a multitude of locally adapted populations, particularly across spatially heterogeneous environments (Fraser, Weir, Bernatchez, Hansen, & Taylor, 2011). In marine ecosystems, the coastal zone represents a particularly important example of habitat heterogeneity. There is increasing evidence from coastal marine environments that species can evolve local adaptations to their respective environmental conditions (Conover & Present, 1990; Sanford & Kelly, 2011; Sjöquist, Godhe, Jonsson, Sundquist, & Kremp, 2015). Gene flow, however, common in the marine environment, is expected to erode genetic differences not linked to spatially heterogenous selection. If so, remaining genetic differences should reflect local adaptations (Bernatchez, 2016; Savolainen, Lascoux & Merilä, 2013; Tigano & Friesen, 2016).

Little is known about the spatial and temporal scales of local adaptations in coastal areas (Conover, Clarke, Munch, & Wagner, 2006). Inter‐annual variability in physical factors (e.g., temperature, currents, freshwater run‐off, sea ice—all of which affect the timing/availability of food), superimposed on long‐term climatic trends, challenges adaptive strategies in coastal populations. Additional challenges to adaptation and genetic integrity of coastal populations arise from introgression between differently adapted conspecific populations, including populations inhabiting the open ocean (cf Lenormand, 2002; Tigano & Friesen, 2016).

Detecting local adaptations in the wild is challenging, especially in aquatic environments where animals are not easily observed throughout their life. However, it might still be possible to measure important components of fitness, such as individual growth and survival (e.g., Hendry & Stearns, 2004). In particular, juvenile growth rate can shape the adult life stages and reproductive success through its influence on age and size at maturation, survival and longevity (Hutchings, 1993). Typically, faster juvenile growth correlates with maturation at a younger age and larger body size (Alm, 1959). Larger individuals are also more fecund and may have higher survival rates in natural environments (Fernández‐Chacón, Moland, Espeland, & Olsen, 2015; Oosthuizen & Daan, 1974). That said, human‐induced selection imposed by harvesting will often target the faster growing large individuals and thereby change the fitness landscape in favour of slower growing small phenotypes (Olsen & Moland, 2011; Swain, Sinclair, & Hanson, 2007).

Recent genomic studies on Atlantic cod (*Gadus morhua*) have contributed to a novel perspective on population genetic structure in marine waters. In coastal areas, genetic structure in this species has repeatedly been found to be dominated by the presence of two genetically distinguishable types that appear to coexist during a large part of the life cycle. In the north‐east Atlantic, this takes the form of the presence in coastal waters of migratory (termed north‐east Arctic cod or NEAC) and stationary (Norwegian coastal cod, NCC) populations (Berg et al., 2016; Kirubakaran et al., 2016; Sarvas & Fevolden, 2005; Westgaard & Fevolden, 2007). A similar sympatry of Atlantic cod types is seen in Icelandic waters (Thorsteinsson, Pálsson, Tómasson, Jónsdóttir, & Pampoulie, 2012), in the North Sea along the Skagerrak coast (Barth et al., 2017; Sodeland et al., 2016 and below) and in the western Atlantic (Barney, Munkholm, Walt, & Palumbi, 2017). Thus, instead of genetic divergence being primarily a reflection of multiple, local populations maintaining genetic differentiation through partial isolation, the largest component of genetic divergence reflects the presence of only two (or at least just a few) genetically divergent types that coexist throughout the coastal area in varying proportions.

Examples of sympatric population structuring have frequently been reported also in freshwater fishes, typically salmonids, lake‐stream sticklebacks, lake whitefish and smelts (Vuorinen, Bodaly, Reist, Bernatchez, & Dodsen, 1993; Taylor, 1999; Moser, Roesti, & Berner, 2012). As an example, long‐term coexistence of sympatric, yet genetically distinct, populations of sticklebacks from lakes and tributary streams with divergent life histories (Moser et al., 2012) is found on both sides of the Atlantic. Food niche separation and size differences among fish from different habitats have been discussed as barriers to gene flow that most likely stem from historical isolation (Moser et al., 2012). Further, there are also examples of cryptic population divergence without any morphological differences, such as populations of brown trout (*Salmo trutta*) within small mountain lakes (Andersson, Johansson, Sundbom, Ryman, & Laikre, 2016; Palmé, Laikre, & Ryman, 2013; Ryman, Allendorf, & Ståhl, 1979).

The finding of sympatric populations opens many questions related to the issue of creation and maintenance of intraspecific biodiversity. For example, What are the biological mechanisms allowing closely related conspecific populations to coexist, apparently for a long time? One part of this issue pertains to mechanisms for reproductive isolation; another to ecological and potentially adaptive differences between populations. This study addresses the latter. Combining habitat and environmental data, juvenile growth and genetic monitoring of juvenile cod along a 500‐km long coastline over 14 years, we document (i) the stable coexistence of two genetically differentiated types or populations of Atlantic cod in coastal areas at large (~100 km) and exceedingly small (~10 m) geographic scales, and (ii) consistent growth differences between the two cod types across all environmental factors. These findings are discussed in relation to the biological relevance of intraspecific differentiation, potential mechanisms for coexistence and implications for management.

## 2 MATERIALS AND METHODS

### 2.1 Study species and area

The Atlantic cod has a wide geographic distribution in the North Atlantic, from Cape Hatteras to Disco Bay in the west and from Bay of Biscay to the Barents Sea in the east. The species also inhabits brackish waters of the Baltic Sea and Arctic lakes with intermittent connections to the sea (Hardie, Gillett, & Hutchings, 2006; Hardie et al., 2008). It is one of the world’s commercially most important fish species and in need of improved coastal management (Svedäng, Stål, Sterner, & Cardinale, 2010). By virtue of its commercial value, the Atlantic cod’s life cycle, ecology, physiology and genetics have been the subject of considerable research, and there currently are available extensive genomic tools for this species, including a reference genome (Star et al., 2011; Tørresen et al., 2017).

Atlantic cod display a range of reproductive strategies. Migratory oceanic cod may perform long‐distance migration to spawning areas, where eggs and larvae are transported with ocean currents to the nursery/feeding grounds. Cod inside fjords or bays, often named coastal cod, tend in contrast to be more stationary and complete their entire life cycle within a restricted geographic area (Canada, Gilbert Bay: Green & Wroblewski, 2000; Iceland: Pampoulie, Storr‐Paulsen, Hovgaard, Hjörleifsson, & Steinarsson, 2011; Skagerrak: Rogers, Olsen, Knutsen, & Stenseth, 2014). Cod may spawn and release more than one million eggs per kilogram of somatic body weight under good nutritional conditions (Wroblewski, Hiscock, & Bradbury, 1999). Pelagic eggs hatch within three weeks following which they remain in the water column and feed on zooplankton until they settle to the bottom as 3–5 cm demersal juveniles.

Along the Norwegian Skagerrak coast, the Norwegian Institute for Marine Research (IMR) is responsible for one of the longest annual time series of fish abundance in the world (from 1919 to present). This unique survey uses a standardized protocol, employed during the months of September and October, covering more than 130 locations (“stations”) where annual beach seine hauls are conducted (Olsen, Carlson, Gjøsæter, & Stenseth, 2009). The beach seine survey samples a large number of fish species (Barceló, Ciannelli, Olsen, Johannessen, & Knutsen, 2016), including young (mostly 0‐group) Atlantic cod.

**Figure 1:**
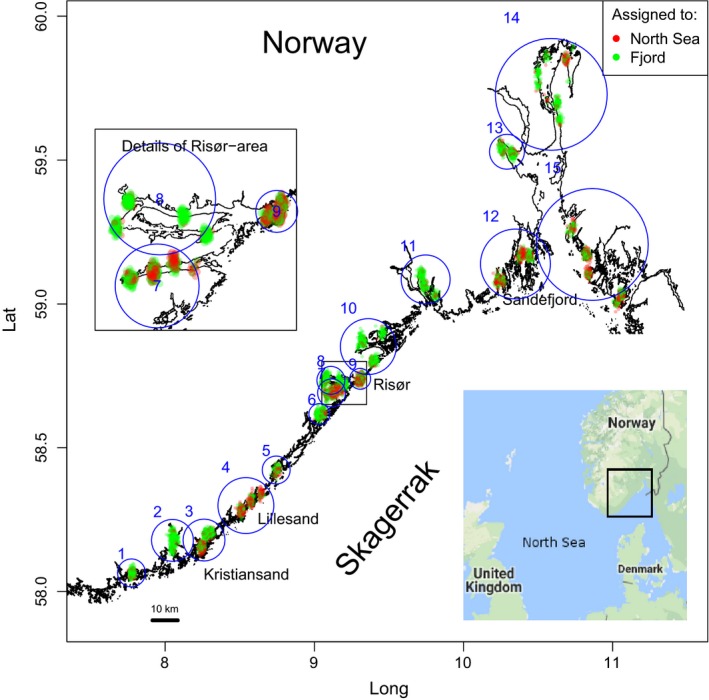
Study area along the Norwegian Skagerrak coast. Position and genetic assignments of all 6,383 individual cod are indicated with coloured dots (red = North Sea type; green = fjord type; positions jittered to minimize overlap). Blue circles indicate the 15 regions (Table 1). Left insert: details of the Risør area. Right insert: location of study area in relation to the North Sea

Previous microsatellite studies of cod samples from the beach seine survey documented persistent genetic differentiation between stations within a fjord and those located among the more exposed, outer skerries (Knutsen et al., 2011). More recently, and employing a much larger number of genetic markers (SNPs), this spatial divergence in coastal cod in the Skagerrak was found to be a reflection of the presence of two different genotype clusters of cod coexisting in various proportions (Barth et al., 2017; Sodeland et al., 2016). One type dominates samples from within fjords whereas the other type is more numerous in outer coastal areas and appears highly similar if not identical to offshore North Sea cod. In a recent study including three fjords within this study area (Kristiansand, Lillesand and Risør: Figure 1), we found no genetic differences among inner fjord populations (Kleiven et al. revised). Hence, inner fjord cod in this region appears as a monophyletic group, distinct from the North Sea cod, and members of the two types can be identified from a small panel of semi‐diagnostic SNPs. Hereafter, we refer to the two types of cod inhabiting coastal Skagerrak as “fjord” and “North Sea” cod, respectively, reflecting their respective areas of dominance (see below). We note that it is still unclear if the latter actually were spawned and hatched in the North Sea and subsequently transported to the Skagerrak coast by ocean current (Knutsen et al., 2004; Stenseth et al., 2006) or if they represent demographically separate outer coastal population(s) that are influenced by gene flow from the North Sea.

### 2.2 Sampling

Sampling of young‐of‐year (0‐group) cod for this study utilized the aforementioned annual survey and included 14 sampling years between 2000 and 2015 (Tables 1 and 2) from fixed positions (survey stations) along the Norwegian Skagerrak coast (Figure 1). The survey involves about 130 beach seine hauls annually, each covering approx. 700 m^2^. During each haul, we note the habitat type and coverage, using a water binocular. Juvenile cod were put in a cooler and frozen whole on‐board within 30 min of sampling. At a few localities where stations were geographically very close (less than a few hundred metres apart), cod were pooled and frozen as one sample. Some stations were not collected in all years. Upon arrival at the laboratory, individuals where assigned a unique ID, collected for otoliths, measured for length (fork length), and fin clipped for DNA extraction.

**Table 1:** Sample regions, number of beach seine stations (N stations), total number of 0 +  cod caught (N fish) and estimated frequency of these that were found to be of putative North Sea origin (Freq.NS), as well as fish length statistics (mean and SD of North Sea and fjord assigned fish, and for the total catch), over the period from 2000 to 2015

Region	N.stations	N.fish	freq.NS	Length.NS (±*SD*)	Length.fjord (±*SD*)	Length.tot (±*SD*)
1 Torvefjord	3	323	0.25	12.14 ± 2.31	9.96 ± 1.52	10.50 ± 1.99
2 Topdalsfjord	7	577	0.20	12.57 ± 2.54	9.69 ± 1.92	10.03 ± 2.20
3 Lillesand	8	620	0.37	10.59 ± 2.31	8.82 ± 2.05	9.48 ± 2.31
4 Grimstad	5	491	0.60	11.67 ± 2.51	9.77 ± 2.12	10.92 ± 2.54
5 Flødevigen	3	115	0.36	11.04 ± 1.71	8.74 ± 2.18	9.58 ± 2.30
6 Tvedestrand	4	297	0.19	10.31 ± 1.93	8.51 ± 1.49	8.86 ± 1.74
7 Sandnesfjorden	8	478	0.42	11.67 ± 2.51	9.56 ± 1.85	10.45 ± 2.40
8 Nordfjorden	8	401	0.11	11.58 ± 2.11	8.56 ± 1.72	8.89 ± 2.00
9 Risør skerries	4	467	0.51	12.43 ± 2.55	10.57 ± 2.56	11.53 ± 2.72
10 Kragerø	11	387	0.09	11.09 ± 2.51	8.64 ± 1.72	8.87 ± 1.94
11 Grenland	8	315	0.16	11.55 ± 2.48	8.26 ± 1.65	8.80 ± 2.18
12 Hvasser	13	596	0.59	10.30 ± 2.42	8.88 ± 2.14	9.72 ± 2.41
13 Holmestrand	7	402	0.20	9.45 ± 1.60	8.08 ± 1.44	8.36 ± 1.57
14 Oslofjord	18	319	0.15	11.83 ± 2.60	9.24 ± 2.27	9.64 ± 2.51
15 Hvaler	19	695	0.56	9.25 ± 1.71	8.16 ± 1.52	8.77 ± 1.72
Total	126	6,483	0.35	10.90 ± 2.51	9.00 ± 1.99	9.67 ± 2.36

**Table 2:** Sample year, number of stations (N.stations), number of individuals genotyped (N.genotyped), number of individuals not assigned (na), number of individuals assigned to the North Sea (NS) or fjord (fjord), frequency of individuals assigned to the North Sea (freq.NS) and length of each genetically assigned group and length of the total material

Year	N.stations	N.genotyped	Assigned to			Freq.NS	Length.NS (±*SD*)	Length.fjord (±*SD*)	Length.tot (±*SD*)
			na	NS	Fjord				
2000	68	816	4	178	634	0.22	11.43 ± 2.33	9.28 ± 1.65	9.75 ± 2.03
2001	43	259	0	98	161	0.38	11.67 ± 2.55	8.83 ± 2.46	9.90 ± 2.85
2003	44	1038	6	404	628	0.39	10.39 ± 2.02	8.62 ± 1.83	9.31 ± 2.09
2004	21	98	0	44	54	0.45	12.50 ± 3.26	9.95 ± 2.82	11.10 ± 3.27
2005	11	167	2	70	95	0.42	12.33 ± 2.29	9.38 ± 2.08	10.63 ± 2.61
2006	17	397	1	249	147	0.63	9.22 ± 1.63	8.37 ± 1.51	8.90 ± 1.64
2007	37	782	6	132	644	0.17	9.46 ± 2.22	8.00 ± 1.41	8.24 ± 1.67
2008	40	166	0	48	118	0.29	11.77 ± 3.00	8.90 ± 2.19	9.73 ± 2.77
2009	27	409	8	151	250	0.38	13.01 ± 2.97	10.68 ± 2.81	11.61 ± 3.09
2010	20	437	9	82	346	0.19	13.03 ± 2.35	9.78 ± 1.79	10.37 ± 2.27
2011	11	825	13	441	371	0.54	10.02 ± 1.89	9.39 ± 2.27	9.76 ± 2.07
2013	49	415	2	100	313	0.24	11.64 ± 2.75	8.20 ± 1.46	9.04 ± 2.37
2014	34	191	48	30	113	0.21	10.42 ± 1.76	9.54 ± 2.18	9.72 ± 2.12
2015	44	483	1	211	271	0.44	11.89 ± 2.33	9.47 ± 1.68	10.53 ± 2.32
Total		6,483	100	2,238	4,145	0.35	10.90 ± 2.51	9.00 ± 1.99	9.67 ± 2.36

### 2.3 Genetic analyses

As an aid in classifying individual cod to type origin, we used reference samples from fjords and the North Sea. The reference sample representing the offshore population comprised individuals collected as mature adult cod in the North Sea (two positions, in the central and the north‐eastern North Sea, respectively) and the reference sample representing the fjord was juvenile (0+) cod from the most sheltered innermost areas of three fjords (Kristiansand, Lillesand, Risør) located within this study area (cf. Figure 1).

Genomic DNA was extracted from 6,483 juvenile coastal cod samples, using a Viogene Inc. miniprep system, and genotyped with a Sequenom MassARRAY multiplex of 40 SNPs, specially developed by to distinguish among individuals of the fjord and North Sea types as described below. In brief, a total of 9,187 SNPs from 10k SNP array were initially scored on a subset of the present material (see Sodeland et al., 2016). Based on these scorings, SNPs were ranked by Nei’s (1973) *G*_ST_ between inner fjord and North Sea samples and filtered based on linkage disequilibrium to exclude SNPs that were highly linked. SNPs with a composite linkage disequilibrium (CLD; Gao et al. 2008) >0.5 to a higher ranked SNPs were excluded. After filtering, 40 high‐ranked SNPs were selected for genotyping in the multiplex. In the present material, only 26 of the 40 SNPs in the multiplex could be scored successfully, and these 26 SNPs were subsequently used for genetic assignments. Subsequent mapping of the 26 SNPs to the Atlantic cod reference genome (Star et al., 2011) revealed that one of these SNP (ss1712301578) was positioned within the recently reported inversion on Atlantic cod linkage group 7, while four (ss1712298913, ss1712300848, ss1712301111 and ss1712303294) were positioned within the inversion on linkage group 12 (Sodeland et al., 2016) whereas the remainders were distributed throughout the genome (cf. Figure S1). Accessions for these SNPs in the dbSNP database (Sherry et al., 2001) are given in Table S1. All assignments of coastal cod to the two reference samples were carried out with the Geneclass II software (version 2.0, Piry et al., 2004). Individuals with fewer than 20 scored SNPs or with a resulting assignment score (i.e., likelihood ratio) lower than 80% were excluded from further consideration, leaving 6383 individuals for the final analysis.

### 2.4 Otolith growth analyses

Sagittal otoliths were prepared for analysis of growth based on their daily increments to examine whether different ages or growth rates best explain differences in size (fork length) between individual juvenile cod. Six otoliths from each of the two cod types in Lillesand and seven otoliths from each type in Sandefjord (cf. Figure 1) were analysed from fish caught during 19–23 September 2015 in the two areas.

After transverse sectioning, otolith photographs were taken at 100 times magnification, except at the core, which was photographed at 400 times magnification to make it easier to observe and measure the narrow increments. The best photographs were chosen for daily increment counting and measuring; these decisions were based on clarity and sharpness, while making sure the chosen ones overlapped, so no increments were lost. All measurements were taken in “analySIS” (SIS, GMBH).

Increment number (i.e., age) and width were used to calculate both hatching date and growth rate. The daily growth rate was calculated using the biological intercept method (Campana & Jones, 1992). The biological intercept used was corresponding to fish and otolith radius at hatching and equalled 4.5 mm (*L*_*i*_) and 9 μm (*O*_*i*_), respectively. The length of the fish at a given age is given by:

(1) $$L_{a}=L_{c}+\left(\left(O_{a}-O_{c}\right)*\left(L_{c}-L_{i}\right)\right)/\left(O_{c}-O_{i}\right)),$$

where *L*_*a*_ is length at age *a*,* L*_*c*_ is length at capture, *L*_*i*_ is the fish length at the biological intercept, *O*_*a*_ is otolith radius at age *a*,* O*_*c*_ is otolith radius at capture and *O*_*i*_ is the otolith radius at the biological intercept. The daily growth corresponding to a single increment can then be calculated as:

(2) $$\Delta L=L_{t+1}-L_{t}=\left(\left(L_{c}-L_{i}\right)/\left(O_{c}-O_{i}\right)\right)*\left(O_{t+1}-O_{t}\right)$$

Differences in observed length of juvenile fish arise due to differences in age/hatching date or growth rates. Using the otolith‐based age and growth estimates from the 26 individuals, we asked the question whether larger fish are larger because they are older or because they have grown faster. First, we compared ages, lengths and growth rates among populations and locations, using a two‐way ANOVA. To examine which of the two predictors (age and growth rate) explained most of the variation in the observed length of the aged fishes, irrespectively of population and location, we used linear models

(3) lm(Length(mm)∼age(days)+growth rate(mm/day))

The explained deviance by each predictor was estimated using the R package *hier.part* (Walsh & Mac Nally, 2013).

### 2.5 Statistical modelling of individual growth

Linear mixed effects models were used to predict juvenile cod body length in the full data set and using all available data, including environmental and genetic assignment data. Specifically, we asked which factor contributed mostly to juvenile body length (*L*, the response variable). The full model, prior to model selection, included fixed effects of wave exposure (WE), vegetation type (VT), vegetation cover (VC) and cod type or origin (CO, genetic assignment: fjord or North Sea):

(4) $$\begin{array}{cc} L & =c_{0}+c_{1}Y+c_{2}\text{WE}+c_{3}\text{VT}+c_{4}\text{VC}+c_{5}\text{CO}+c_{6}\text{WE}*\text{CO}\\ & \;+c_{7}\text{VT}*\text{CO}+c_{8}\text{VC}*\text{CO} \end{array}$$

where *c*_0_ is the intercept. Two‐way interaction effects between cod type (CO) and the three environmental covariates (WE, VT and VC) were included to evaluate if any fitness differences between the two cod types depended on nursery habitat type, thereby testing the hypothesis of micro‐habitat‐related selection as a possible explanation for the coexistence of genetically divergent types. In addition, year (Y) was modelled as a factor capturing annual variation in environmental effects not measured by us (e.g., temperature and food availability). Wave exposure (WE) was modelled as a linear effect. Vegetation type (VT) was modelled as a factor with two levels defined as the dominant flora within the beach seine stations: (i) eel grass and (ii) macroalgae. Vegetation cover (VC) was modelled as a linear effect ranging from 1 (sparse vegetation) to 5 (fully vegetated substrate). Data were estimated from each beach seine station as follows. Wave exposure was taken as the significant wave height (in m) calculated based on fetch and decadal records of wind measurements from representative meteorological stations (source Norwegian Meteorological Institute, eklima.met.no). Wave exposure, vegetation type and vegetation cover were defined for each beach seine station and assumed to be constant among years and averaged over years. Individual cod origin was modelled as a factor with two levels: 0 (fjord) and 1 (North Sea). Beach seine sample stations were grouped into 15 regions based on geographic proximity and shoreline features (Rogers et al., 2011; Table 1). We included region in the model as a random effect to account for the fact that samples within each region might not be independent. Models were fitted using the R package *nlme* (Pinheiro, Bates, DebRoy, & Sarkar, 2017). Fish length and wave exposure were log‐transformed to stabilize the variance. The original data set contained information about a total of 6,504 juvenile cod. Statistical modelling of fish length was based on a subset of 5,730 individuals, primarily because some fish samples were pooled across beach seine stations which prevented us from determining the appropriate environmental covariates for these individuals and partly because some individuals did not meet the criteria for genetic assignments (above).

Model selection was performed in two steps, by first determining the appropriate random structure and then searching for the most parsimonious fixed structure (Zuur, Ieno, Walker, Saveliev, & Smith, 2009). In the first step, we compared a model containing the random effect of region as well as the full suite of fixed effects to a simpler model without the random effect (retaining all of the fixed effects). Model selection was based on the Akaike information criteria, AIC (Burnham & Anderson, 2002), and restricted maximum likelihood (REML) estimation. A residual plot indicated that the full model including a random effect fits the data adequately. In the second step, the fixed effects were sequentially removed and model selection based on AIC, using a maximum likelihood (ML) estimation procedure. Inference was based on the most parsimonious model, and in this final step, parameter estimation was based on a REML procedure.

## 3 RESULTS

During 14 years between 2000 and 2015, a total of 11,019 juvenile cod were caught at the 126 beach seine stations in the survey (Tables 1 and 2). We retained 6,484 of these for genetic analyses with the SNP panel, resulting in 6384 individuals being scored successfully, that is, genotyped for at least 20 loci and with an *Geneclass II* assignment score of 80% or higher (Tables 1 and 2). Of the 100 unscored individuals, 55 failed because of technical problems (i.e., poor DNA quality, zero or very few loci genotyped) and 45 others received a *Geneclass II* score below 80% and were excluded from further consideration. Of the 6,384 successfully assigned individuals, 4,146 assigned to the fjord reference sample thus indicating a fjord type, and 2,238 assigned to the North Sea reference sample (Table 2) with a putative North Sea origin. The fjord type thus dominated numerically the total sample, with an average proportion of 64.9%.

### 3.1 Distribution of cod type over time and space

**Figure 2:**
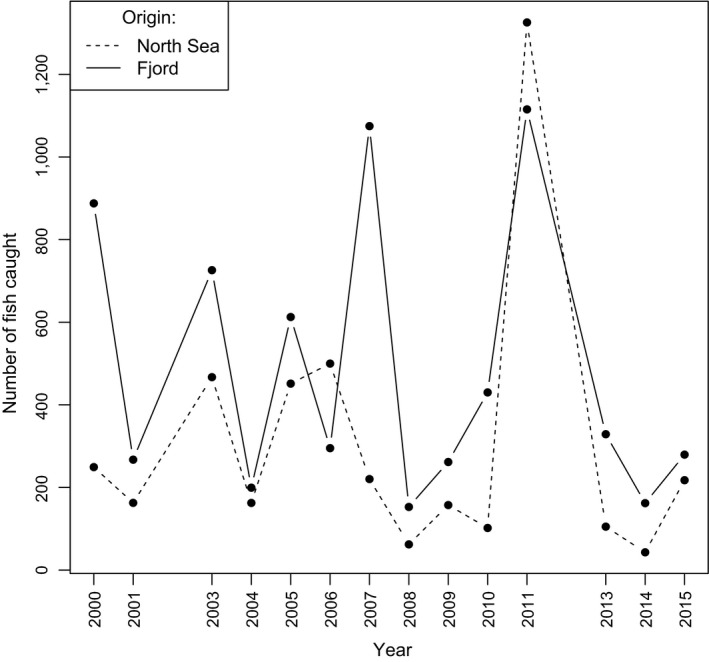
Estimated numbers of cod of putative North Sea (dashed line) and fjord origin (solid) over years in the beach seine survey, that is the whole coastline. Estimates were calculated from Table 2 as the products of N_tot and Freq_NS and 1‐Freq_NS, respectively

**Figure 3:**
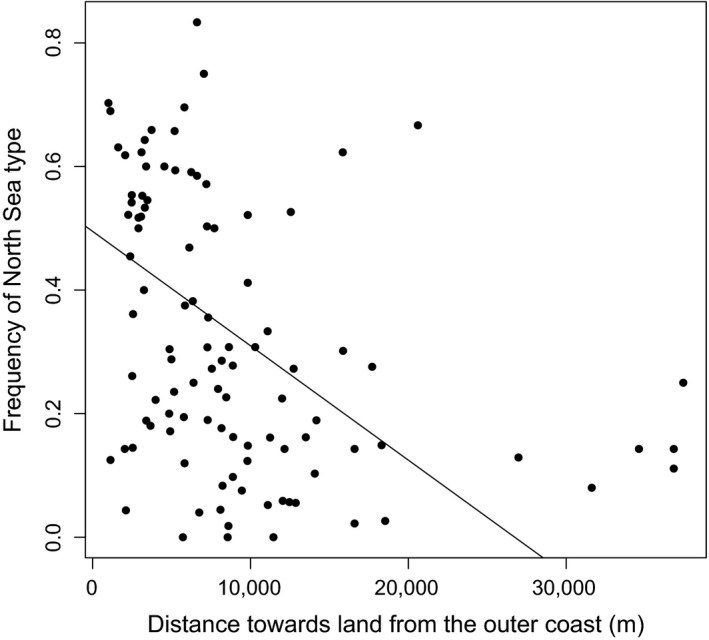
Frequency of North Sea cod type in 102 sample stations relative to their distance towards land from the outer coast, i.e., a hypothetical line bordering the outer skerries (sample stations with fewer than 5 cod excluded). The solid line represents the weighted (by sample size) linear regression (slope = −1.85*10^−5^, *P *=* *6.8*10^−7^)

The frequency of cod of fjord or North Sea type varied among regions (Table 1) and years (Table 2; Figure 2). The two types tended to fluctuate in numbers together over years, with some notable exceptions. In the strong year of 2011, with a total catch of 2,441 individuals, 54% of the 820 genotyped individuals assigned to the North Sea (Table 2), whereas in another strong year (in 2007), less than 20% of genotypes cod were estimated to be of North Sea origin. There is also substantial variation among the 15 geographic regions, with proportions of putative North Sea cod varying from 13% to 62% (Table 1) on average over years. Low proportions of North Sea cod seem to characterize a few inner fjords, as expected, but this tendency is far from universal. Instead, both types co‐occur in all sampled regions throughout the study area. Further, both types were found at 94% of the beach seine stations over the years, and 81.3% of all seine hauls (i.e., station and year) that had more than five individual cod (267 of 455 hauls) included members of different origins (Table 3). On average, there was a probability (Simpson’s diversity index) of 29.2% that two randomly drawn cod from the same beach seine haul were of different origins. These observations verify the long‐term coexistence of both cod origins down to the smallest sampling scale (haul level, ca. 700 m^2^). While largely occurring together, there was a strong tendency for higher proportions of the fjord type in sample localities located farther inland of the outer coast line (defined as a hypothetical line bordering the outer skerries), that is, deeper inside fjords (Figure 3).

**Table 3:** Co‐occurrence of two cod types (“fjord” and “North Sea”) at large and small sampling scales. The table shows the type diversity (i.e., probability of two random individuals being of different types) and proportions of samples with both cod types presents. Four different sample levels are depicted: total coastline, geographic region (groups of proximate sample stations: blue circles in Figure 1), beach seine station and individual beach seine haul (i.e., station and year: only one haul was taken from each station each year)

Level (number of samples)	Cod type diversity^a^ (*SD*)	Proportion of both types^b^
Total (1)	0.450	1.0
Region (15)	0.380 (0.121)	1.0
Station (125)	0.347 (0.148)	0.941
Haul (455)	0.292 (0.175)	0.813

a Simpson's index of diversity (Simpson, 1949). Calculated as 1—freq.NS^2^—(1‐freq.NS)^2^, and averaged over samples (weighted by sample size). SD is the (weighted) standard deviation.

b Excluding samples with <6 individuals (excludes 23 of 125 stations and 188 of 455 hauls).

### 3.2 Otolith growth patterns

Considering the total data set (2000–2015), age‐0 juvenile cod length ranged from 5.2 to 23.9 cm with a mean of 9.67 cm (Table 1). On average, age‐0 individuals with a putative North Sea origin were larger (10.90 cm, with an *SD* of 2.51 cm) than cod with a fjord origin (9.00 ± 1.99 cm).

**Figure 4:**
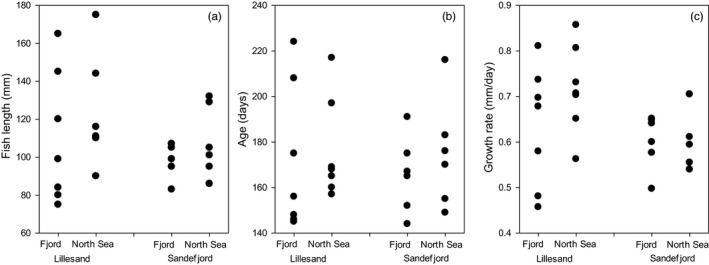
Body length (a), age (b) and otolith‐based estimate of growth rate (c) of 26 juvenile fjord (*n* = 13) and North Sea (*n* = 13) cod from Lillesand (stations 36–39 and 41; *n* = 14) and Sandefjord (stations 214 and 218; *n* = 12) caught during 19–23 September 2015

Based on daily otolith increment counts, in the subset of 26 individuals (13 NS and 13 fjord cod) subjected to such analyses, juvenile cod ages ranged between 144 and 224 days, corresponding to hatching dates between 5 February and 3 May (2015) (Figure 4). The range of hatching dates was similar between all combinations of origin and location, and no significant differences were found (two‐way ANOVA, *F*_2,23_ = 0.358, *p* = .7). Average daily growth rates varied between 0.46 to 0.86 mm/day with an overall mean across all individuals of 0.65 mm/day and the differences were not significant (two‐way ANOVA, *F*_2,23_ = 2.44, *p* = .11). In addition, there was no significant difference in fish length in these restricted samples (two‐way ANOVA, *F*_2,23_ = 2.04, *p* = .15). To examine the contribution of hatching date, that is age, and growth rates to the observed variation in length of the juvenile cod, we employed hierarchical partitioning of the deviance in the data. Overall, the model (Eq. (1)) explained 99.3% of the variation in fish length, with 52.8% explained by growth rate and 47.2% explained by hatching date. Hence, variation in growth rate was marginally more important than hatching date in explaining the variability in length of the 26 juvenile cod examined.

### 3.3 Factors affecting individual growth

The mixed effects model strongly supported the inclusion of a random structure to explain fish length (Eq. (4)), compared to a model for which fish body length was explained from fixed effects only (ΔAIC = 104, cf. Table 4 for model selection). A total of 8% of the variance in cod body length was associated with the random effect of sampling region. When considering the fixed structure, model selection supported the inclusion of year, wave exposure, flora type, flora cover and cod origin for explaining body length. Model selection also supported the inclusion of two‐way interaction effects between (i) cod origin and wave exposure and (ii) cod origin and vegetation type. Removing the latter interaction effect increased the AIC value by only 1.4 units, so the support for this interaction effect was moderate. Removing the first interaction effect increased AIC by 3.7 units, so the support for the interaction effect between cod origin and wave exposure was strong. Simpler models removing any of these predictors, as well as more complex models including the interaction effect between cod origin and vegetation cover, all had higher AIC scores (Table 4).

**Table 4:** Comparison of linear mixed models for predicting age‐0 Atlantic cod body lengths (*L*). Showing the fixed part of the model structure and the Akaike information criterion (AIC) of each model. Sampling year (Y), cod genotype (CO, fjord or North Sea type), wave exposure at each sampling location (WE), bottom vegetation type (VT, eel grass or macroalgae) and bottom vegetation cover (VC) were included as fixed effects. In addition, the region of capture was included as a random effect (not shown). The most parsimonious model selected for inference is shown in bold

Model structure	AIC
*L* = Y + WE*CO + VT*CO + VC*CO	−3039.4
***L*** = Y + VC + WE*CO + VT*CO	−**3040.6**
*L* = Y + VC + VT + WE*CO	−3039.2
*L* = Y + VC + WE + VT*CO	−3036.9
*L* = Y + VC + WE + VT + CO	−3037.1
*L* = Y + WE + VT + CO	−3125.6
*L* = Y + WE + CO	−3064.7
*L* = Y + CO	−2977.8
*L* = Y	−1867.6

**Figure 5:**
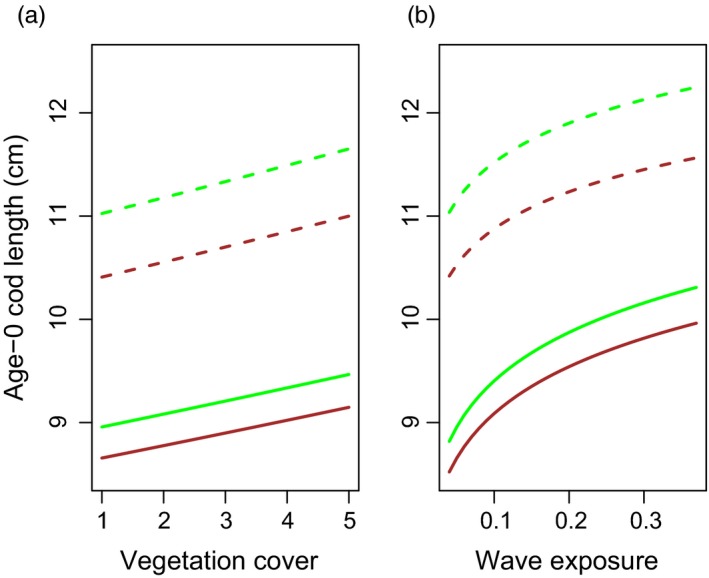
Body length of North Sea (dashed lines) and fjord cod (solid lines) as predicted from the most parsimonious linear mixed model (see Results), showing (a) predictions for eelgrass (green) and macroalgae (brown) habitats at increasing vegetation cover (1 = no vegetation, 5 = fully vegetated habitat) and mean wave exposure, and (b) eelgrass and macroalgae habitats at increasing wave exposure and mean vegetation cover. Note that the North Sea cod type has a body length approx. 2 cm longer than the fjord

From the above considerations, inference about cod body size variation, as a component of variation in fitness, was based on the most parsimonious model, containing a random effect of sampling region as well as fixed effects of year, wave exposure, vegetation type, vegetation cover and cod type, including two‐way interaction effects between (i) cod type and wave exposure and (ii) cod type and vegetation type (parameter estimates in Table 5). In any given year and region, the model predicted that age‐0 cod should be larger at stations with more bottom vegetation cover and higher wave exposure (Figure 5). Predicted body lengths were also larger in eelgrass habitats (Figure 5) compared with macroalgae habitats. Moreover, predicted body lengths were almost 2 cm larger for age‐0 North Sea cod than for fjord cod over all wave exposures (Figure 5a) and habitats (Figure 5b). The interaction term between cod type and vegetation type indicated that the size difference between age‐0 North Sea cod and age‐0 fjord cod was larger in eelgrass habitats compared with macroalgae habitats. The interaction effect between cod type and wave exposure indicated that the size of age‐0 North Sea cod changed less across sites with different wave exposure compared with the size of age‐0 fjord cod (cf. Figure 5).

**Table 5:** Parameter estimates with standard errors (*SE*) for the fixed effects included in the model selected for inference about variation in age‐0 cod body length. The initial year of sampling (2000), eelgrass vegetation and cod of the fjord type were coded as zero in the model (reference levels)

Model term	Parameter estimate	*SE*	*p*‐Value
Intercept	2.355	0.035	<.0001
Year_2001_	−0.040	0.014	.0044
Year_2003_	−0.050	0.009	<.0001
Year_2004_	0.143	0.023	<.0001
Year_2005_	−0.017	0.017	.3333
Year_2006_	−0.103	0.013	<.0001
Year_2007_	−0.120	0.010	<.0001
Year_2008_	0.016	0.016	.3383
Year_2009_	0.092	0.013	<.0001
Year_2010_	0.058	0.012	<.0001
Year_2011_	−0.119	0.011	<.0001
Year_2013_	−0.072	0.012	<.0001
Year_2014_	0.016	0.018	.3777
Year_2015_	0.040	0.011	<.0001
Wave Exposure	0.070	0.010	<.0001
Vegetation_Macroalgae_	−0.034	0.010	.0003
Vegetation cover	−0.014	0.004	.0001
Origin_NorthSea_	0.149	0.026	<.0001
Wave Exposure*Origin_NorthSea_	−0.023	0.010	.0170
Vegetation_Macroalgae_*Origin_NorthSea_	−0.023	0.013	.0648

## 4 DISCUSSION

The present study directly addresses the question of what might constitute an appropriate spatial scale that reflects different adaptive responses by Atlantic cod to environmental change. We have documented two genetically distinct cod types, likely to have divergent origins, coexisting across a range of nursery habitats during 14 years of standardized sampling along coastal Skagerrak. Based on their genetic and phenotypic differences, we conclude that fjord‐type and North Sea‐type constitute two distinct but coexisting ecotypes of coastal cod, the latter possibly originating from offshore North Sea spawning. Comparing microhabitats, both ecotypes were larger in areas that were (i) vegetated rather than barren, (ii) dominated by eel grass rather than algae and (iii) more exposed to the open ocean. However, genetic background played a major role in determining individual length and explaining nearly 2 cm of size difference, representing an average difference of 20% in length and 80% in body weight between ecotypes.

Stable coexistence of genetically divergent populations of the same species within the same habitat appears to be infrequent in broadcast‐spawning marine fish. While other examples of coexisting populations are known, these typically involve temporal mixing of adult populations at feeding grounds, for example, Atlantic herring (*Clupea harengus*) (Ruzzante et al., 2006) and Atlantic cod in Greenland (Therkildsen et al., 2013). Here, we find that the ecotypes coexist at the finest possible sampling scale both spatially and temporally, that is, at the level of individual beach seine haul covering no more than 700 m^2^ of habitat (Chan et al., 2003). Also, the coexistence is seen to extend to the juvenile stage and appears pervasive throughout the coastal zone. With both cod ecotypes present in the majority of beach seine hauls, true coexistence at the same site and time is verified, suggesting that segregation into different microhabitats does not explain their stable coexistence.

Although it is clear that the Skagerrak coastal cod ecotypes differ genetically, it is not clear whether these differences can account for the consistently, and substantively, larger body sizes of the North Sea ecotype. Based on a limited number of otolith samples obtained from two of the 15 sampling regions (Lillesand, Sandefjord), we find no differences in hatching date, suggesting that the two ecotypes might be of similar age and, therefore, have different growth trajectories. The back‐calculated estimates of hatching dates (late January until early May) and average daily growth (0.39–0.92 mm/day) correspond well with previous work on North Sea and coastal cod (Fey & Linkowski, 2006; Nielsen & Munk, 2004). Based on the results of a separate study, Roney et al. (submitted) found no differences in spawning time between cod sampled from the inner and outer waters of the same fjord (Risør), again suggestive of similarity in hatching date. In one of the two regions where otoliths were examined (Lillesand), we also find evidence of faster growth among cod of the North Sea ecotype, a finding consistent with the hypothesis that ecotypic differences in individual growth have a genetic basis. Recent analyses (Barth et al., 2017; Sodeland et al., 2016) have identified several large polymorphic chromosomal variants in coastal cod within our study area, where the frequency of each variant largely correlates with genetic origin (Sodeland et al., 2016). These variants may play a role in maintaining adaptive genetic differences between cod ecotypes.

### 4.1 Ecological and evolutionary mechanisms

The stable spatiotemporal coexistence of divergent cod genotypes raises several testable hypotheses as to what is responsible for their maintenance. Given the existence of spawning individuals (Roney et al., 2016), eggs (Ciannelli et al., 2010; Knutsen et al., 2007) and larvae in coastal waters (Ciannelli et al., 2010), it is clear that at least one of the ecotypes reproduces in Skagerrak. Given the geographic origin of the fjord ecotype, it is reasonable to conclude that fjord cod spawn in sheltered coastal Skagerrak waters and that their ability to do so reflects some degree of local adaptation. Thus, it is the presence of the North Sea ecotype that requires explanation. This ecotype might spawn regularly in Skagerrak, or be transported there during early life, or both (André et al., 2016). Dispersal of the North Sea ecotype during the egg/larval/early‐juvenile stages into Skagerrak is highly probable, given the speed and location of regional oceanic currents (Knutsen et al., 2004; Stenseth et al., 2006). This would account for their presence as 0‐ and 1‐year‐olds in coastal waters. There would not seem to be any physical or biological barriers that would prevent the North Sea ecotype from spawning in parts of Skagerrak, such as the outer skerries. This then raises the question as to whether the 0‐ and 1‐year‐old North Sea ecotype competes with, or has a higher fitness than, the fjord ecotype.

Our limited otolith analyses indicate that North Sea cod *can* have higher growth rates than local fjord‐type cod. Whether this growth difference is widespread is not known and should be analysed with a larger dataset. One thing that is clear, however, is that the North Sea cod ecotype is considerably larger than the fjord ecotype independently of habitat and that size differences among individual juvenile cod primarily are explained by growth differences. This might be a phenotypic response to higher food supply in outer waters and it might have a genetic component (as noted above). The linear mixed modelling revealed a strong effect of ecotype after accounting for known environmental factors such as vegetation type, vegetation cover and wave exposure, and also after accounting for annual variation in the general environment. This suggests that the size difference between the two ecotypes could be partly genetic.

Size‐at‐age in fish can be a major component of fitness (Hutchings, 1993). Therefore, the persistent coexistence in the same habitats of the presumably subordinate smaller type suggests the presence of trade‐offs with other fitness components and balancing selection on somatic growth rate (Billerbeck, Lankford, & Conover, 2001). Given the asymmetric distribution with respect to distance from the outer coastline (Figure 3), one possible explanation is that the smaller fjord type is better adapted to particulars of the inshore or fjord environment. Potential adaptive mechanisms might include different energy requirements in exposed vs. sheltered environments, size‐related differences in feeding, and differences in salinity, temperature or oxygen level tolerances (Berg et al., 2015).

### 4.2 Implications

Initiated to monitor coastal Atlantic cod, the Skagerrak beach seine survey has been ongoing since 1919 (Barceló et al., 2016). Genetic information obtained from fish sampled in this survey allowed us to differentiate fjord and North Sea cod ecotypes and to describe elements of their spatiotemporal dynamics in coastal waters. Their coexistence in nursery habitats (present study), and probably also later as adults (Barth et al., 2017), has management implications. As one example, our observation that the proportional representation of the fjord ecotype can exceed that of the North Sea ecotype among juvenile cod in some years draws attention to the need to acknowledge the potentially substantive contributions that the fjord ecotype can make to the recruitment of cod to the coastal fishery.

Generally speaking, a fundamental challenge to successful management and conflict resolution is to correctly identify the spatial scale at which strategies for harvesting, conservation and climate‐change mitigation are developed. Atlantic cod in coastal Skagerrak (within 12 nautical miles off the coast) are currently managed as a single unit along with cod along the Norwegian coast up to 62°N (a distance of approximately >1,000 km). In contrast, our work provides clear evidence that young cod in coastal Skagerrak represent a mix of probable local fjord populations and a component from offshore (North Sea) population(s).

Mixed‐”stock” fisheries that do not include risk‐averse measures to protect genetic sub‐units (e.g., ecotypes) that differ in abundance, productivity and(or) resilience can result in the overfishing or recovery inhibition of the smaller units (Bonanomi et al., 2015). Similarly, the effectiveness of strategies to conserve coastal biodiversity will be limited if fundamental elements of genetic and ecological variability, such as that represented by ecotypes, are excluded. Thus, fishery management plans and other mitigation strategies that do not account for ecotypic variability are unlikely to meet objectives related to the sustainability of fish and fisheries.

## DATA ARCHIVING STATEMENT

Data available from the Dryad Digital Repository: https://doi.org/10.5061/dryad.hm249b5.

## AUTHOR CONTRIBUTION

The study was conceived and designed by H.K., P.E.J. and E.M.O. Assessment of genotypes and data quality was performed by H.K, P.E.J. and M.S. Otolith analysis was performed by P. G., K.‐E.M.J. Oceanographic modelling was carried out by J.A. Samples were provided by H.K. and E.M.O. The manuscript was written by H.K. with contributions from P.E.J., J.H. and E.M.O. All authors read and revised the manuscript.

## CONFLICT OF INTEREST

None declared.
