# Supplementary material for: Stable coexistence of genetically divergent Atlantic cod ecotypes at multiple spatial scales
Source: Evol Appl. 2018 May 17;11(9):1527–39. doi: 10.1111/eva.12640 (PMC6183466; doi:10.1111/eva.12640)
Supplement: Supplementary file 1 [file EVA-11-1527-s001.docx]

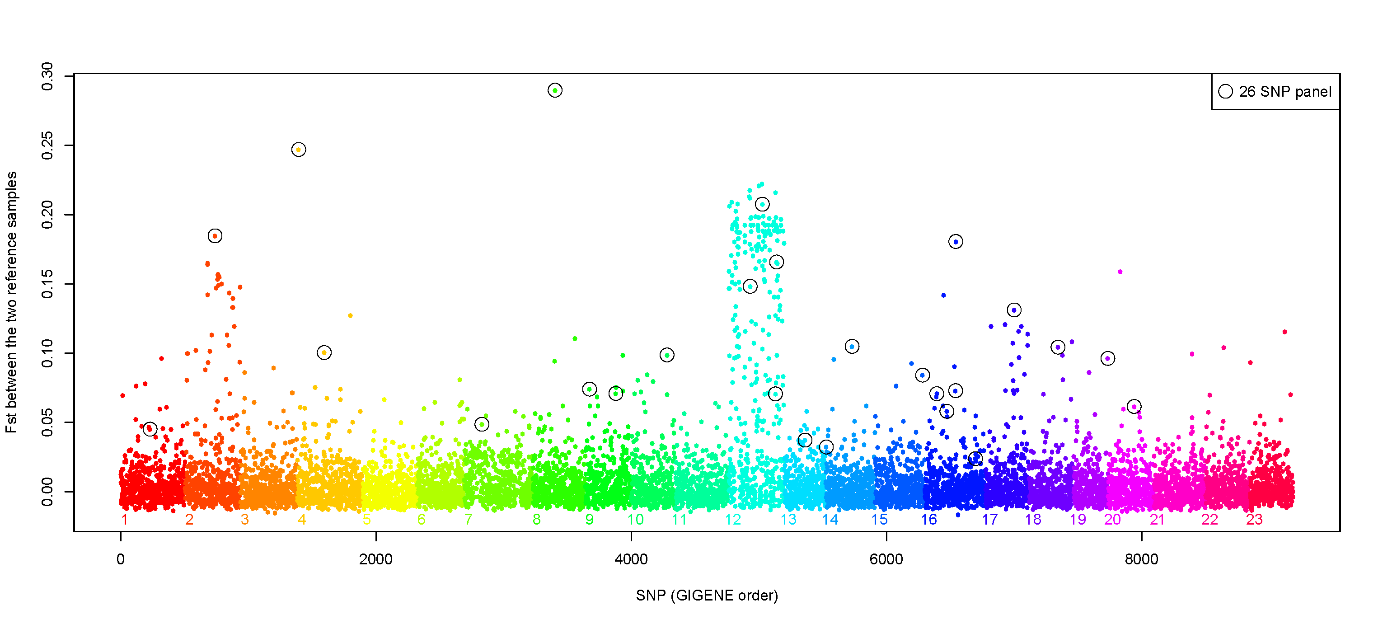


Figure S1

Manhattan plot of genetic divergence (*F*_ST_) between the two reference samples, from Skagerrak inner fjords and from the North Sea, for 9187 SNPs from a 10K SNP chip (data from Ring et al.), mapped to the cod genome assembly (version 1: Star et al. 2011) using linkage information from Sodeland et al. (2016, Supplementary Informatuion). Numbers and colors refer to different linkage groups (i.e., chromosomes). The 26 SNPs used in the present study are encircled (see Table S1 for details).
