# Supplementary material for: Stable coexistence of genetically divergent Atlantic cod ecotypes at multiple spatial scales
Source: Evol Appl. 2018 May 17;11(9):1527–39. doi: 10.1111/eva.12640 (PMC6183466; doi:10.1111/eva.12640)
Supplement: Supplementary file 2 [file EVA-11-1527-s002.docx]

Table S1. SNP accessions and linkage group (LG) number for the 26 SNPs used for genetic assignments.

| **SNP** | **LG** |
| --- | --- |
| ss1712303563 | 1 |
| ss1712297946 | 2 |
| ss1712295890 | 4 |
| ss1712296565 | 4 |
| ss1712297081 | 6 |
| ss1712301578 | 7 |
| ss1712297159 | 8 |
| ss1712299108 | 9 |
| ss1712301593 | 9 |
| ss1712300625 | 10 |
| ss1712298913 | 12 |
| ss1712300848 | 12 |
| ss1712301111 | 12 |
| ss1712303294 | 12 |
| ss1712297476 | 13 |
| ss1712302534 | 14 |
| ss1712303465 | 14 |
| ss1712297702 | 15 |
| ss1712297985 | 16 |
| ss1712300777 | 16 |
| ss1712300779 | 16 |
| ss1712302403 | 16 |
| ss1712303675 | 16 |
| ss1712298141 | 18 |
| ss1712297319 | 19 |
| ss1712298954 | 20 |
